# Software Framework for Controlling Unsupervised Scientific Instruments

**DOI:** 10.1371/journal.pone.0161671

**Published:** 2016-08-29

**Authors:** Benjamin Schmid, Wiebke Jahr, Michael Weber, Jan Huisken

**Affiliations:** 1 Max Planck Institute of Molecular Cell Biology and Genetics, 01307 Dresden, Germany; 2 Optical Imaging Centre Erlangen, Friedrich-Alexander-University of Erlangen-Nuremberg, 91054 Erlangen, Germany; 3 Harvard Medical School, Boston, Massachusetts 02115, United States of America; 4 Morgridge Institute for Research, Madison, Wisconsin 53715, United States of America; Imperial College London, UNITED KINGDOM

## Abstract

Science outreach and communication are gaining more and more importance for conveying the meaning of today’s research to the general public. Public exhibitions of scientific instruments can provide hands-on experience with technical advances and their applications in the life sciences. The software of such devices, however, is oftentimes not appropriate for this purpose. In this study, we describe a software framework and the necessary computer configuration that is well suited for exposing a complex self-built and software-controlled instrument such as a microscope to laymen under limited supervision, e.g. in museums or schools. We identify several aspects that must be met by such software, and we describe a design that can simultaneously be used to control either (i) a fully functional instrument in a robust and fail-safe manner, (ii) an instrument that has low-cost or only partially working hardware attached for illustration purposes or (iii) a completely virtual instrument without hardware attached. We describe how to assess the educational success of such a device, how to monitor its operation and how to facilitate its maintenance. The introduced concepts are illustrated using our software to control eduSPIM, a fluorescent light sheet microscope that we are currently exhibiting in a technical museum.

## Introduction

Science outreach can be performed in different ways, e.g. through web publishing, public discussions, school visits or dedicated outreach events. A more exploratory and interactive possibility that is particularly well suited for conveying technical advances in scientific instrument development is the exhibition of new instrument prototypes to the public, e.g. in museums.

Oftentimes it is possible to use the hardware of a laboratory prototype more or less unchanged for a public exhibition. The software, however, comprises the interface to the user and must account for the altered role of the instrument. Modern scientific devices are highly complex, and scientists typically receive special training to operate the software. In contrast, visitors in a museum with diverse educational backgrounds explore the instrument by trial. Unlike in a laboratory environment, it is the software’s responsibility to provide an attractive user interface and guarantee robust, non-stop operation with only little or no supervision.

In this paper we elaborate the concepts of a modular software framework that is well suited for controlling an autonomously running scientific instrument. Our software focuses on user-friendliness and elaborate error handling. It recovers automatically from errors, originating, e.g. from the communication with the hardware. If a severe outage prohibits correct functioning, our software keeps providing a seemingly functioning instrument by simulating normal operation. The introduced framework allows one to operate a fully functioning device, but can at the same time be used to simulate normal function if mock hardware is attached for illustration purposes only, or in the complete absence of hardware.

The concepts introduced here can generally be used to control unsupervised scientific devices, perhaps operated at locations that are difficult to access, e.g. at contaminated areas, in deep ocean or for speleology. As an example, we provide a specific implementation that is used to control eduSPIM [1], an educational light sheet fluorescence microscope (or Selective Plane Illumination Microscope, SPIM [2]) that we have developed recently for an interactive exhibition in a technical museum, to support the UNESCO International Year of Light 2015 [3].

## Framework for operating real hardware, mock hardware or a purely virtual instrument

Thorough error handling is critical for the unsupervised operation of a fully functional instrument with working hardware attached to it. Otherwise, malfunctioning hardware or errors during the communication with peripherals would immediately lead to software crashes and leave the system unusable. To recover from this, the administrator would be forced to check frequently to ensure continuous functioning.

Here, we introduce an elaborate error handling design that allows the software to recover from unforeseen situations. We propose a modular framework that defines an interface for each attached peripheral. For example, a camera interface defines functions for setting the exposure time, starting the acquisition and reading the acquired image data. These interfaces are implemented by individual software modules that handle the communication to the corresponding peripheral, typically using the manufacturer’s application programming interface (API). At the same time we implement a second module for each interface that simulates its operation. In the camera example, the simulating implementation loads and uses pre-acquired data from the hard-drive.

**Fig 1:**
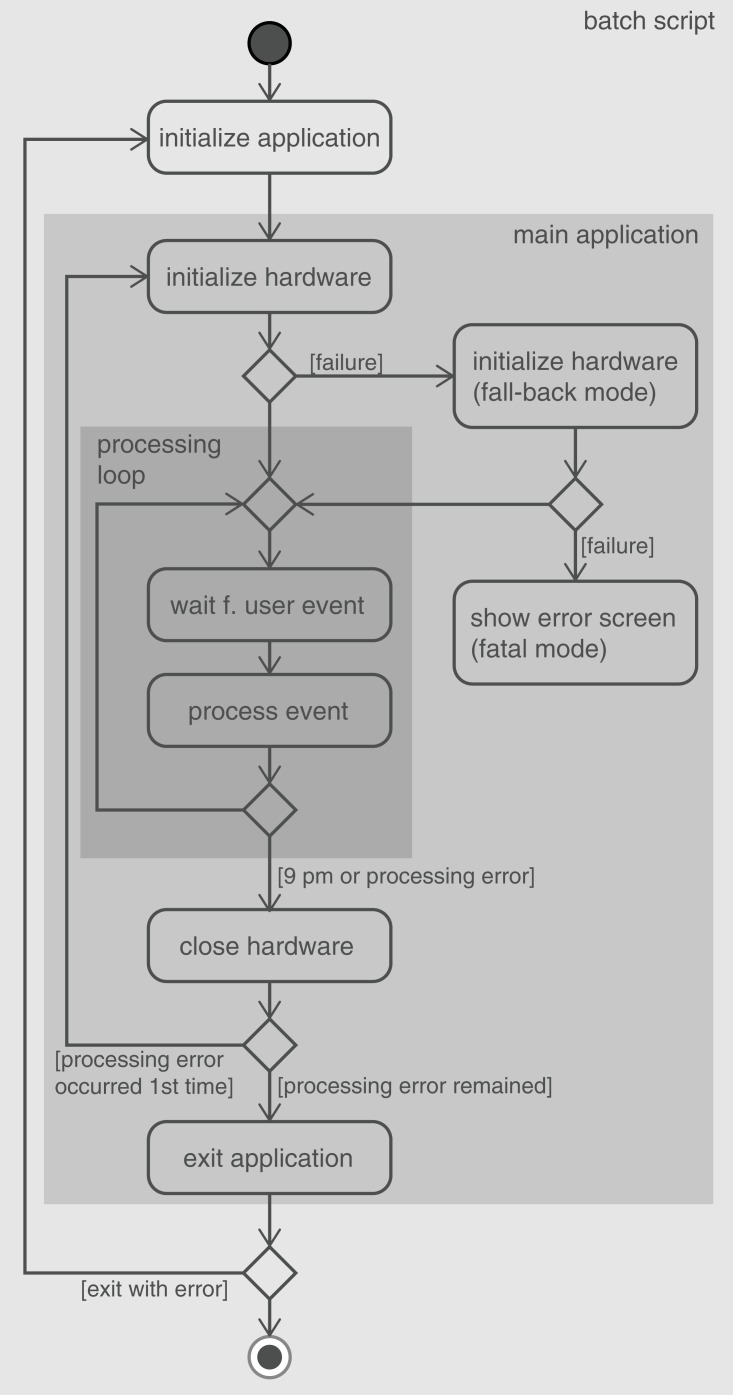
Error handling during initialization and normal operation. The main program was started from a Windows batch script in a loop. If any error occurred during hardware initialization, the software switched to fall-back mode, which simulated normal operation but used a pre-acquired data set. If switching to fall-back mode also failed, the software entered fatal mode, and an error message was displayed. Otherwise, the program waited for the visitor to press one of the buttons, upon which the corresponding functionality was executed. If any error occurred, all hardware devices were closed and re-initialized. If the error persisted, the application exited with an error code. Back in the batch script, the application was re-started as long as the exit code indicated an error.

The software uses the real implementation for each device during normal operation and without any error occurring. In case of a failure, communication to all devices is closed and re-initialized. If the error persists, the software exits completely to make sure that all hardware resources are released properly. To provide ongoing operation in such a case, the software is started from a batch script repeatedly from inside a loop. In case it exits with an error code indicating a hardware problem, it is restarted automatically (Fig 1). If any error occurs during startup, the software switches to the simulating versions of all modules, thereby providing a seemingly functional instrument. In case an error occurs that renders the instrument completely unusable (e.g. user input cannot be gathered any more), even the simulating versions won’t allow a usable device, and an emergency screen notifies users about the problem and asks for their patience until the device has been repaired.

If the software’s purpose is the control of an educational instruments, e.g. an exhibition in a museum or school, there may only be limited resources for the necessary maintenance service. Our software can be be started explicitly in simulating mode, so that the exhibition can continue without disruption until maintenance is possible.

Pursuing this idea further, it is even possible to exhibit an instrument with only mock hardware or without any hardware attached to it at all. Only the software needs to be installed, which keeps costs and efforts minimal. Our framework checks autonomously for attached hardware. If none is available, it automatically downloads a pre-acquired example data set. It is therefore straightforward to replicate the exhibit numerous times and use it practically anywhere, e.g. in school lessons or at special events.

## Computer setup

A number of considerations regarding computer setup and configuration are important for guaranteeing continuous availability of an unsupervised instrument.

We set up the PC’s BIOS to boot the computer at a fixed time early every morning. We put a shortcut to our software into the Startup folder under the Windows start menu, so that our software starts automatically after booting. It switches immediately to full-screen mode to hide the operating system from users and to focus their attention to the instrument’s software. Our software closes itself at a fixed time every evening, well after any expected usage. Additionally we configured the Windows Task Scheduler to shut down the computer during the night, to save power and freshly initialize the connections to the peripherals every day. To guarantee that no other application window (such as an update notification of the operating system) obscures our software during normal usage times, we implemented a tool for putting the main window of our application into foreground, using the Windows API. This tool was called at a regular interval of 5 seconds from within our software.

We decided for a mini PC (Intel NUC), because the space available in the museum was limited, and we wanted to hide the computer and all hardware controllers inside the table to not distract the visitors from the optical setup. As an operating system we are using Microsoft Windows 7 64-bit professional.

## Monitoring and maintenance

For monitoring and remote maintenance of an unsupervised instrument, the computer needs to be connected to the Internet, possibly via WLAN. Our proposed software solution uses a cloud service (Dropbox, https://www.dropbox.com) to synchronize folders with our computers in the lab. Information about any exception that occurred is not only sent to an administrator via email, but also saved and archived to a synchronized folder. Additional emails on successful starts and shut downs inform the administrator about the integrity of the system. For trouble-shooting and occasional maintenance such as installing security updates for the operating system, we used a screen-sharing software (TeamViewer, https://www.teamviewer.com) to log in remotely. In contrast to related software, TeamViewer does not require the computer to have a public IP address, so that the computer can reside behind a router outside of our institute’s local network.

## Usage statistics and website

To assess the success of a remotely operated instrument, it is important to collect and visualize information about its usage and the data acquired by it. We used the logging framework SLF4j (http://www.slf4j.org) to log not only exceptions, but also all actions triggered by the user to a synchronized folder. In another synchronized folder, we regularly saved and archived renderings of the instrument’s output. We analyze, visualize and publish these data on a dedicated website, which is dynamically updated in regular intervals using JavaScript, AJAX and jQuery (https://jquery.com).

**Fig 2:**
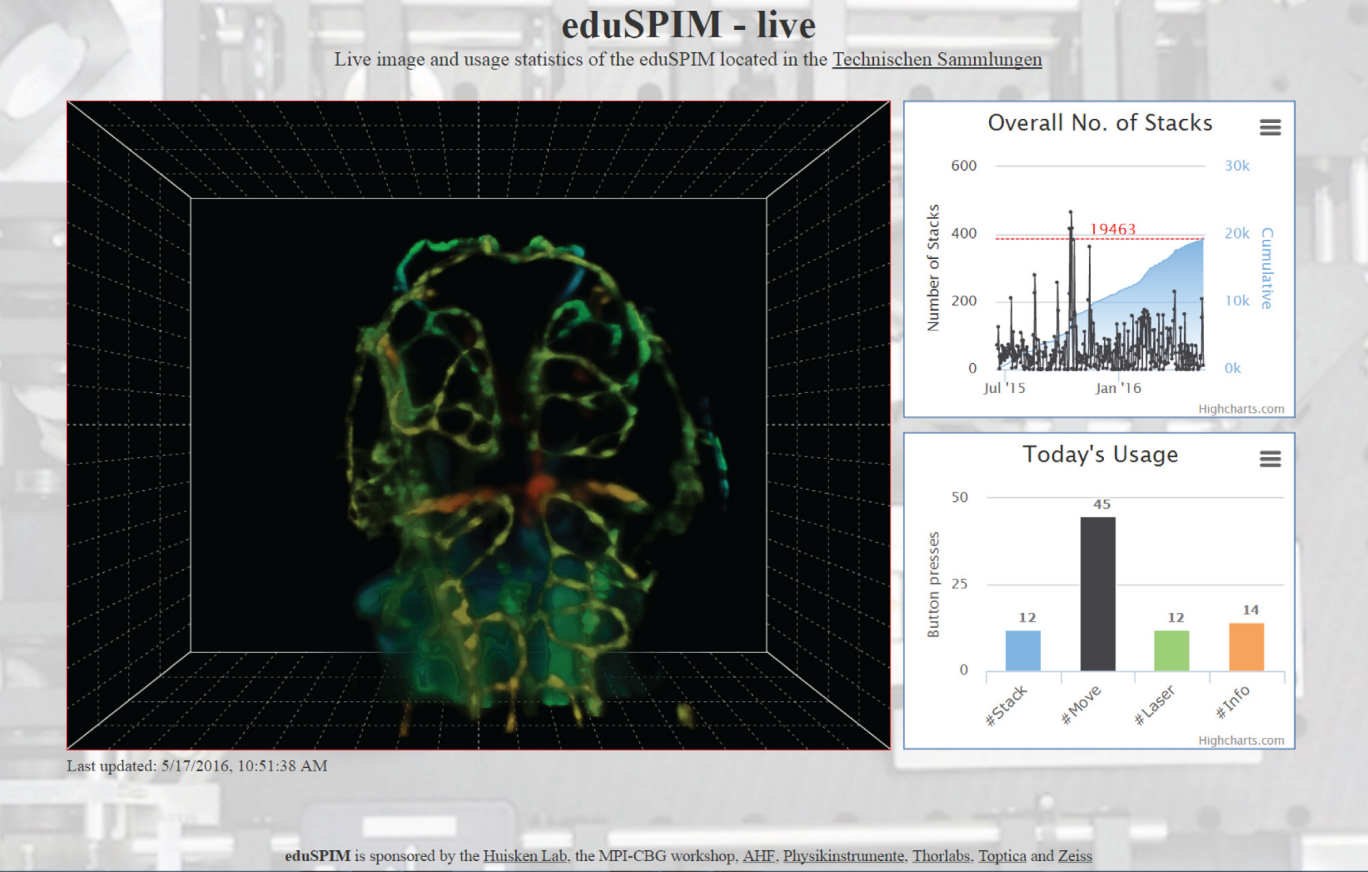
Screenshot of the eduSPIM website, http://www.eduspim.org. The page was divided into three panels: the main panel containing a snapshot of the latest rendering displayed on the microscope computer (left), a diagram showing the number of stacks acquired since the exhibition was opened (upper right) and a histogram indicating how often each button was pressed on the current day (lower right). All panels were dynamically updated using JavaScript. The data shown here were acquired in stack mode. The fluorescence signal was rendered using a combination of volume ray casting and depth colour coding.

For eduSPIM, we save stack renderings in a publicly shared folder. To limit the amount of data we only keep one rendering per day. Furthermore, we save the most recently acquired image and a table with the number of times each button was pressed per day. The accompanying website (http://www.eduspim.org, Fig 2) shows the latest snapshot, a diagram with the number of stacks acquired since the opening of the exhibition and a histogram with the distribution of button presses on the current day. Our website was implemented such that it updated itself automatically and could be displayed on public screens, e.g. in the lobby of our institute. To limit data transfer, only the small table file was fetched at a fixed interval of 5 seconds, using JavaScript, AJAX and jQuery (https://jquery.com), and checked for modification. The diagrams were updated and the latest snapshot image was downloaded and displayed on the page only if necessary.

## An intuitive interface for users

The user interface consists of both the controls for the user to interact with the system, as well as a visual representation of the instrument’s results. Generally, the functionality offered to the user and the input controls should be kept minimal and concentrate on the key aspects. If the instrument is located at a museum, this is essential to attract the visitors’ attention and to convey its educational message. For a scientific instrument operated under extreme conditions, it is important to be intuitively usable. In both cases, controls such as buttons must be robust, under certain conditions they may also need to be water- or fireproof, or they may need to be operable with gloves. Although input controls can be implemented in software, hardware solutions are preferable to avoid the need of a keyboard or mouse and thereby prevent users from interacting with the operating system. To connect the hardware to the PC and read its state from within the control software, some kind of input/output (I/O) interface is required, e.g. a data acquisition device. Sometimes the signal sent by the hardware needs additional filtering, e.g. to avoid flickering when pressing a button, in which case we recommend to use a microcontroller board.

**Fig 3:**
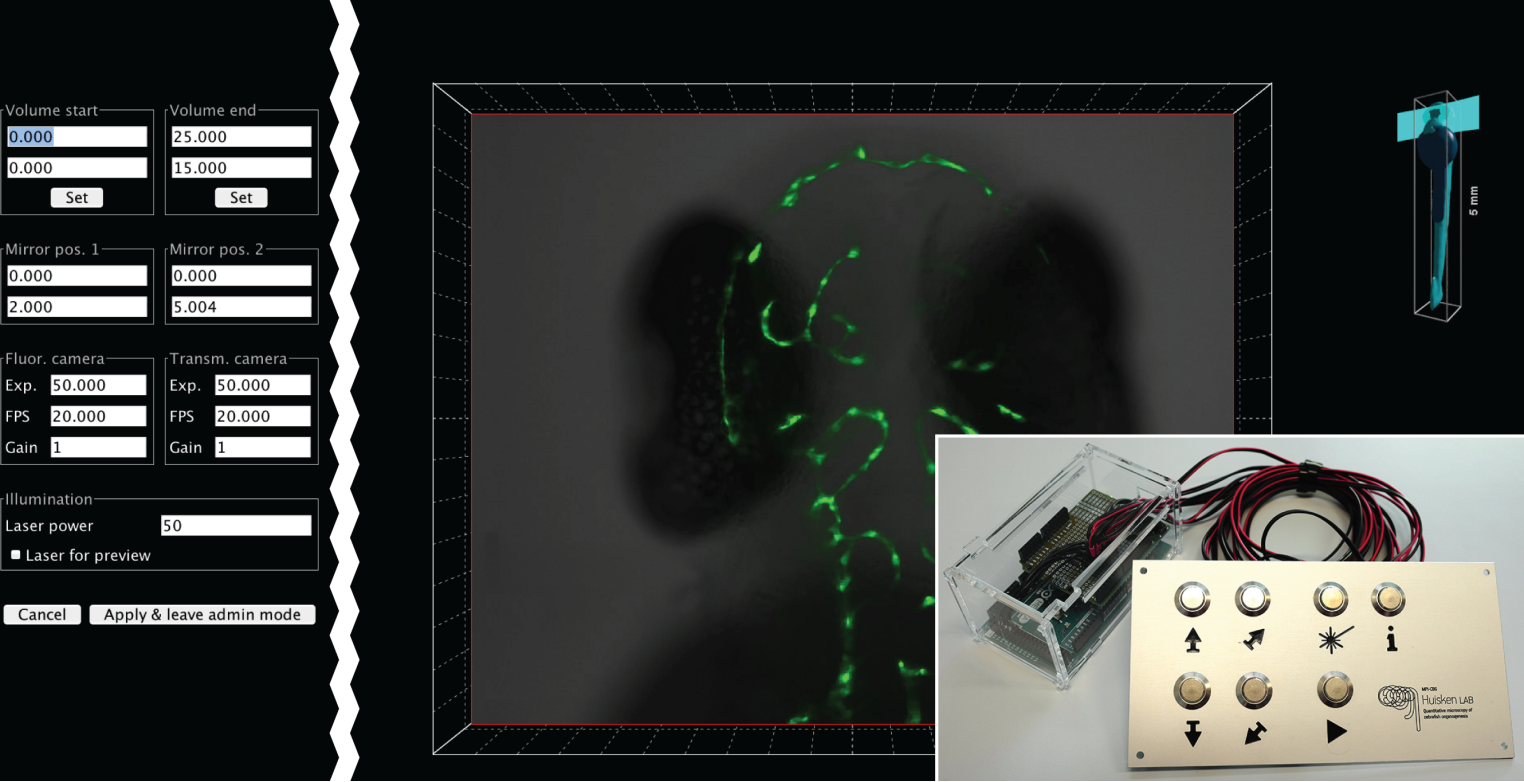
User interface. During normal operation, the user interface is clean and simple. The computer screen displays only a rendering of the acquired microscope data (centre), together with a small 3D model of the zebrafish sample indicating the current imaging plane (upper right). The data shown here were acquired during preview mode. The transmission image was rendered as an opaque surface in grey, overlaid with the fluorescence signal from the vasculature in green. A rendering of the data acquired in stack mode is shown in Fig 2. A plate with seven buttons (lower right), connected to the control computer via an Arduino board, lets the visitors move the sample forward and backward, record a stack, manually switch the laser on or display a screen with additional information. For maintenance, e.g. when the sample is exchanged, we implemented an additional panel (left) to adjust numerous settings conveniently. This panel is hidden during normal operation and only displayed via a keyboard shortcut by an administrator.

For eduSPIM, we designed a control panel with seven buttons (Fig 3): two for positioning the sample along the vertical axis, two for adjusting the imaging plane from dorsal to ventral, and one button each for recording a stack, for switching the laser manually on and for displaying an information screen. We connected the buttons to the computer via an Arduino board (https://www.arduino.cc, S1 Fig). Custom software on the Arduino took care of unwanted flickering.

Visual representation of the instrument’s results depend very much on the kind of data that is obtained. In general, the representation needs to be as simple as possible to be easily comprehensible.

In the laboratory, data acquired by scientific instruments is usually processed post-acquisition. Processing and analysis steps are tailored to an experiment and aim to confirm or disprove a scientific hypothesis, typically in a quantitative manner. In contrast, a standalone instrument, either in an exhibition or in the field, has a fixed purpose with a given processing and visualization pipeline. If the instrument has an educational purpose, an illustrative representation of the data that best demonstrates its functionality is essential. If it is an imaging device such as a microscope, the acquired images need to be rendered in a way that the imaged structure can easily be recognized. This is particularly important in case of three-dimensional imaging. At the same time, processing and rendering must be efficient to be applicable in real-time for immediate display.

For eduSPIM we implemented a preview mode that was active while the user moves the sample manually through the light sheet, and a stack mode that captures a three-dimensional *z*-stack at the current position. In both modes, the display was continuously updated while images were obtained from the cameras. To support the visitor in navigating through the imaged sample, a small 3D model of it was displayed next to the rendering, indicating the light sheet position and the current imaging plane (Fig 3). In preview mode, the transmission image was rendered in grey, overlaid with the fluorescence signal using a green lookup table. Scaling the images corresponding to the current axial imaging position and placing them into a coordinate system provided a 3D impression. In stack mode, we combined basic volume ray casting with depth colour coding for real-time 3D rendering. We pre-calculated lookup tables with a different colour for each plane. As in preview mode, the display was continuously updated as images were acquired. Each pixel in an image was given an RGB value according to the corresponding lookup table and a transparency value proportional to the square of its intensity. Images were then scaled depending on their *z* position and overlaid from back to front, taking into account their transparency values (Fig 2). This was accomplished in real-time without the need for sophisticated graphics hardware using Java2D drawing functions.

## An augmented interface for maintainers

For a standalone instrument to be intuitively usable, the functionality exposed to users is typically limited. Normally, it is however necessary to adjust several settings from time to time to keep the instrument working optimally. The software needs to provide an augmented interface to maintainers to make these changes. This interface should not be visible to normal users. We implemented a graphical maintenance panel (Fig 3) that was hidden during normal usage but could be made visible by connecting a keyboard and pressing a shortcut. Furthermore, we integrated a panel for entering and executing BeanShell (http://www.beanshell.org) commands. BeanShell is a scripting language that can be embedded into Java applications to execute Java code dynamically at run-time. It provides a flexible way for adding new functionality without a GUI. The BeanShell interface was also hidden during normal operation.

For eduSPIM, we needed to replace the sample regularly, despite an optimized protocol for sample fixation and staining. Upon sample exchange, we adjusted the bounding box of the imaged volume, calibrated the optical elements and fixed acquisition parameters like exposure time and laser power, using the graphical maintenance panel. We used the BeanShell interface to perform non-repetitive tasks, e.g. for time-lapse recording, for recording raw 3D data sets and for automatic acquisition and stitching of multi-tile data sets [4, 5].

## Programming environment

We have implemented our software in Java and C/C++. Java was used for the graphical user interface and the logic flow. C/C++ was used to implement hardware communication to the cameras and motors. The C/C++ routines were called from Java using the Java Native Interface (JNI). Maven (https://maven.apache.org/) was used to build the Java part of the project, while Microsoft NMAKE was used to build the C/C++ modules, using the Microsoft compiler and the Windows 7 SDK. The source code is kept in a Git repository (https://git-scm.com/) for version control and can be publicly accessed on GitHub (https://github.com/bene51/eduSPIM.git). To run the software, no special hardware is needed, since it automatically searches for attached peripherals and, in case of their absence, falls back to the simulating mode.

## Discussion

The concepts presented in this paper apply whenever a scientific instrument needs to be operated reliably without supervision. Our framework focuses on intuitive usage, robustness against hardware failures and ease of maintenance. Its modular design allows to exchange hardware parts easily. To test and validate the concepts we have implemented them in a software framework to control, monitor and efficiently maintain eduSPIM, a light sheet microscope exhibited in a technical museum in Dresden. At the time of writing, it has been continuously running for over nine months, and almost 20,000 stacks have been acquired since the opening of the exhibition.

Although our implementation specifically targets the fully-functional hardware of eduSPIM, it can also run without hardware and imitate normal functionality of a light sheet microscope. It can therefore be used to provide a virtual software-based light sheet microscope on any computer. This can be used to easily demonstrate not only the working principles of light sheet microscopy, but also its application, e.g. to image the vasculature in zebrafish, without the need for any hardware. Such a virtual light sheet microscope on a laptop can be taken anywhere, e.g. to class rooms, conferences, public discussions or it can be used for exhibitions in public locations such as universities or municipal offices, to convey the importance of science for today’s society to the general public and raise the interests of young people in research.

The ideas introduced in this paper are not specific to light sheet microscopes, not even to microscopes in general, and can be used for exhibitions or remote operation of similar devices. Beyond the educational mission, our design can as well serve as a blueprint for unsupervised instruments that are operated at remote locations that might be hard to reach or contaminated.

## Supporting Information

S1 Fig Arduino wiring diagram. For eduSPIM, we connected seven buttons to an Arduino board. Pull-down resistors kept the signal low if a button’s state was open (not pressed). When a button was pressed, the input signal at the corresponding pin switched to high. A change in the buttons’ states was recognized by the software running on the Arduino, which notified the microscope control software on the PC through a serial connection. After a button press, consecutive switches were only allowed after 50 ms delay, to avoid bouncing. Additionally, we used the Arduino for controlling the LED that was used for transmission imaging on the microscope. We automatically switched the LED off if no button was pressed for 5 minutes. We adjusted the LED’s brightness using the LED controller and connected it to the Arduino using a transistor (TIP 120) for switching. We provide the software running on the Arduino on github (https://github.com/bene51/EduSPIM).
(EPS)
